# The N-Formyl Peptide Receptor 2 (FPR2) Agonist MR-39 Exhibits Anti-Inflammatory Activity in LPS-Stimulated Organotypic Hippocampal Cultures

**DOI:** 10.3390/cells10061524

**Published:** 2021-06-17

**Authors:** Ewa Trojan, Kinga Tylek, Monika Leśkiewicz, Władysław Lasoń, Lars-Ove Brandenburg, Marcello Leopoldo, Enza Lacivita, Agnieszka Basta-Kaim

**Affiliations:** 1 Laboratory of Immunoendocrinology, Department of Experimental Neuroendocrinology, Maj Institute of Pharmacology Polish Academy of Sciences, 12 Smętna Str., 31-343 Kraków, Poland; trojan@if-pan.krakow.pl (E.T.); tylek@if-pan.krakow.pl (K.T.); leskiew@if-pan.krakow.pl (M.L.); lason@if-pan.krakow.pl (W.L.); 2 Institute of Anatomy, Rostock University Medical Center, 18057 Rostock, Germany; Lars-Ove.Brandenburg@med.uni-rostock.de; 3 Department of Anatomy and Cell Biology, RWTH Aachen University, 52062 Aachen, Germany; 4 Department of Pharmacy—Drug Sciences, University of Bari, Via Orabona 4, 70125 Bari, Italy; marcello.leopoldo@uniba.it (M.L.); enza.lacivita@uniba.it (E.L.)

**Keywords:** MR-39, formyl peptide receptor 2, hippocampal organotypic cultures, lipopolysaccharide, NLPR3 inflammasome

## Abstract

Accumulating evidence indicates a pivotal role for chronic inflammatory processes in the pathogenesis of neurodegenerative and psychiatric disorders. G protein-coupled formyl peptide receptor 2 (FPR2) mediates pro-inflammatory or anti-/pro-resolving effects upon stimulation with biased agonists. We aimed to evaluate the effects of a new FPR2 ureidopropanamide agonist, compound MR-39, on neuroinflammatory processes in organotypic hippocampal cultures (OHCs) derived from control (WT) and knockout FPR2−/− mice (KO) exposed to bacterial endotoxin (lipopolysaccharide; LPS). Higher LPS-induced cytokine expression and basal release were observed in KO FPR2 cultures than in WT cultures, suggesting that a lack of FPR2 enhances the OHCs response to inflammatory stimuli. Pretreatment with MR-39 abolished some of the LPS-induced changes in the expression of genes related to the M1/M2 phenotypes (including *Il-1β*, *Il-6*, *Arg1*, *Il-4*, *Cd74*, *Fizz* and *Cx3cr1*) and TNF-α, IL-1β and IL-4 release in tissue derived from WT but not KO mice. Receptor specificity was confirmed by adding the FPR2 antagonist WRW4, which abolished the abovementioned effects of MR-39. Further biochemical data showed an increase in the phospho-p65/total p65 ratio after LPS stimulation in hippocampal tissues from both WT and KO mice, and MR-39 only reversed this effect on WT OHCs. LPS also increased TRAF6 levels, which are critical for the TLR4-mediated NF-κB pro-inflammatory responses. MR-39 attenuated the LPS-evoked increase in the levels of the NLRP3 and caspase-1 proteins in WT but not KO hippocampal cultures. Since NLRP3 may be involved in the pyroptosis, a lytic type of programmed cell death in which the main role is played by Gasdermin D (GSDMD), we examined the effects of LPS and/or MR-39 on the GSDMD protein level. LPS only increased GSDMD production in the WT tissues, and this effect was ameliorated by MR-39. Collectively, this study indicates that the new FPR2 agonist efficiently abrogates LPS-induced neuroinflammation in an ex vivo model, as evidenced by a decrease in pro-inflammatory cytokine expression and release as well as the downregulation of NLRP3 inflammasome-related pathways.

## 1. Introduction

Neuroinflammation is a complex multicellular process that plays an important role in the onset and progression of several neurodegenerative and psychiatric disorders [1,2]. Acute inflammation is considered a physiological response to defend the host against pathogens and to maintain homeostasis. Although the acute inflammatory response is protective, dysfunction of its resolution and failure to return to tissue homeostasis lead to tissue damage and chronic inflammation [3,4,5]. Recently, the resolution of inflammation (RoI) was shown to be regulated by specialized pro-resolving mediators (SPMs) [6,7] that are enzymatically derived from essential polyunsaturated fatty acids, including arachidonic acid, eicosapentaenoic acid and docosahexaenoic acid, in a lipoxygenase-dependent manner [8,9]. SPMs exert their biological actions by binding to and activating cognate receptors, of which formyl peptide receptor 2 (FPR2) is of special interest [10,11]. FPR2 (also referred to in the literature as FPRL1 or ALX/FPR2) is a G protein-coupled receptor (GPCR) that binds LXA4 and 15-epi-LXA4 with high affinity. This receptor is expressed on various types of immune cells and in the brain on some neurons, astroglia and microglia [12]. FPR2 is an unconventional receptor not only due to the diversity of its ligands but also because its activation translates into a wide array of biological effects, ranging from pro-inflammatory to anti-inflammatory and pro-resolving effects [13,14,15,16]. FPR2 activation stimulates several signal transduction pathways, depending on the ligand, related concentration and the cell type involved [17]. The ability of FPR2 to mediate opposing effects (pro-inflammatory vs. pro-resolving) is mechanistically related to receptor dimerization, which is induced in a ligand-specific manner [18,19]. Subsequent studies have suggested that the divergent properties of FPR2 may be related to ligand-specific receptor conformational changes associated with downstream signaling. Using a FRET biosensor, the authors of [20] demonstrated that the peptide FPR2 agonist WKYMVm and the lipid agonists LXA4 and ATL induced different receptor conformational changes that resulted in different G-protein activation. Considering that the crystal structure of FPR2 evidenced that the ligand binding pocket is rather large and can accommodate very diverse ligands, it is plausible that each ligand can interact with different amino acids within the binding site, and this can translate in the activation of different signaling pathways [21]. Nevertheless, the role of FPR2 in inflammatory processes remains controversial. Chen et al. [22] showed a decrease in the inflammatory response in FPR2-deficient (FPR2−/−) mice. In contrast, Dufton et al. showed the exacerbation of carrageenan-induced paw edema and passive serum-induced arthritis in FPR2−/− mice [23]. In a mouse model of pneumococcal meningitis, FPR2−/− mice showed a significantly increased glial cell density, whereas immune responses, including the expression of anti-inflammatory cytokines, were decreased [24].

Among the most specific endogenous FPR2 agonists, lipoxin A4 and its analog aspirin-triggered lipoxin (ATL) mainly show anti-inflammatory and pro-resolving profiles [25]. Their pro-resolving roles have been identified in various inflammatory settings, including viral infection [26], and they modulate some processes relevant to acute inflammation, such as leukocyte infiltration, cytokine and chemokine production or the expression of chemokine receptors [27,28,29,30].

Unfortunately, the unfavorable pharmacokinetic properties of LXA4 and its analog represent a limitation for in vivo evaluations and clinical trials of these agonists. Moreover, LXA4 inactivation mostly occurs in microglial cells and involves initial dehydrogenation to 15-oxo-lipoxin A4 [31], limiting the beneficial action of LXA4 in brain tissue.

Recently, we have reported on a series of ureidopropanamide FPR2 agonists with favorable pharmacokinetic characteristics that give a good rationale for testing them as anti-inflammatory and pro-resolving agents. Among these agonists, MR-39 shows good metabolic stability in rat microsomes (t1/2 = 48 min), good passive diffusion and good permeation rates in an in vitro model of the blood–brain barrier. Moreover, our preliminary study showed the ability of MR-39 to suppress pro-inflammatory responses in primary microglial cells [32].

Based on the abovementioned data, in the present study, we decided to investigate the molecular mechanism underlying the potential anti-inflammatory effect of the FPR2 agonist MR39 on bacterial endotoxin (LPS)-exposed organotypic hippocampal cultures derived from control (wild type; WT) and FPR2−/− knock-out (KO) mice. In particular, we evaluated the effects of MR-39 on cell death, nitric oxide release, and the levels of pro- and anti-inflammatory cytokines under both basal and LPS-stimulated conditions. The receptor specificity of the effect of MR-39 was verified using the FPR2 antagonist WRW4. We analyzed the intracellular pathways related to the inflammatory response, including MyD88/TRAF6/NF-кB and inflammasome 3 (NLRP3) signaling, to investigate the mechanisms underlying the effect of MR-39 on organotypic hippocampal cultures obtained from both WT and FPR2−/− mice. Recent data suggested that NLRP3 may be involved in the pyroptosis, a lytic type of programmed cell death in which the main role is played by Gasdermin D (GSDMD). Thus, we also examined the effects of LPS and/or MR-39 on the GSDMD protein level.

## 2. Materials and Methods

### 2.1. Chemicals

((S)-3-(4-cyanophenyl)-N-[[1-(3-chloro-4-fluorophenyl)cyclopropyl]methyl]-2-[3-(4-fluorophenyl)ureido]propanamide)—compound MR-39—was prepared as described in our previous studies [32,33]. The FPR2 antagonist WRW4 was purchased from Alomone Labs, Jerusalem, Israel. The bacterial endotoxin lipopolysaccharide (LPS; Escherichia coli 0111:B4) was obtained from Sigma-Aldrich, St. Louis, MO, USA.

### 2.2. Animals

FPR2-deficient mice (knockout; KO; FPR2−/−) were obtained from Dr. Lars-Ove Brandenburg of the Department of Anatomy and Cell Biology, RWTH Aachen University, Aachen, Germany. Briefly, FPR2−/− KO mice on a C57BL/6 background were generated as described previously [22,24]. The wild type (WT) mice were back-crossed onto the C57BL/6J background for at least five generations. All mice were maintained under standard conditions (a room temperature of 23 °C, 12/12 h light/dark cycle, and lights on at 06:00 a.m.), with food and water available ad libitum. Females were mated with syngeneic male KO or WT mice. All experimental protocols were performed in accordance with guidelines from the Committee for Laboratory Animal Welfare and Ethics of the Maj Institute of Pharmacology, Polish Academy of Sciences, Krakow, Poland.

### 2.3. Establishment of Organotypic Hippocampal Cultures (OHCs)

Six- to seven-day-old WT and KO mouse pups were used to prepare organotypic hippocampal cultures (OHCs) with the method described by Stoppini et al. [34] with slight modifications [35,36]. After decapitation, brains were placed in sterile ice-cold working buffer (96% HBSS, 3.5% glucose, and 0.5% penicillin/streptomycin; all reagents were obtained from Gibco, Waltham, MA, USA). The isolated hippocampi were placed on Teflon discs and cut into 350 µm slices using a McIlwain tissue chopper. Next, slices were transferred to ThinCertsTM-TC Inserts with 0.4 µm pore size membranes (Greiner bio-one, Kremsmünster, Austria) in 6-well plates containing 1 mL of culture medium (containing 50% DMEM + GlutaMax™-I, pH 7.4; 20.5% HBSS; 25% horse serum; 0.1 mg/mL glucose; 1% amphotericin B, 0.4% penicillin and streptomycin; 1% B-27 supplement and HEPES (to maintain pH); all reagents were obtained from Gibco, Waltham, MA, USA). The OHCs were grown in an incubator (37 °C) with an adjustable CO_2_ flow (5%) for 7 days (7 DIV). The OHCs were initiated in regular medium containing 25% horse serum, which was then gradually (from DIV 4th to 7th) tapered to serum-free medium (containing 50% DMEM F-12, pH 7.4; 44% HBSS; 0.1 mg/mL glucose; 1% amphotericin B; 0.4% penicillin and streptomycin, 1% B-27, 1% N-2; and HEPES (to maintain the pH). All reagents were obtained from Gibco, Waltham, MA, USA. The medium was first changed 24 h after culture establishment (half of the volume (0.5 mL)) and then every 48 h (whole volume (1 mL)) thereafter. On the 7th day, the in vitro medium was changed to serum-free medium.

### 2.4. OHC Treatment

On the 7th day, OHCs obtained from WT and KO mice were pretreated with the FPR2 antagonist WRW4 (10 µM) for 30 min. Afterward, MR-39 (1 µM) was added for 1 h, and then OHCs were stimulated for 24 h by adding LPS to the medium (final concentration in the well: 1 μg/mL). Control (unstimulated) OHCs were treated with vehicle (phosphate-buffered saline (PBS)).

### 2.5. Determination of Lactate Dehydrogenase (LDH) Activity

Twenty-four hours after culture stimulation, the level of lactate dehydrogenase released to the culture medium was measured as previously described [35] using a colorimetric method according to the supplier’s protocol (Cytotoxicity Detection Kit, Roche Diagnostic, Mannheim, Germany). The data were normalized to the activity of LDH released from control slices (100%; vehicle-treated WT OHCs) and are reported as a percentage of the control ± SEM (standard error of the mean).

### 2.6. Nitric Oxide (NO) Release Assay

The Griess test was used for the nitric oxide (NO) secretion in the culture medium. According to the protocol, 24 h after stimulation, 50 µL of supernatant were mixed with an equal volume of Griess reagent (Griess A—0.1% N-1-naphthylethylenediamine dihydrochloride and Griess B—1% sulfanilamide in 5% phosphoric acid; Sigma-Aldrich, St. Louis, MO, USA) in a 96-well plate. The absorbance was measured at 540 nm using an Infinite^®^ 200 PRO Detector (TECAN, Switzerland). The data were normalized to the NO released from vehicle-treated cells (100%; vehicle-treated WT OHCs) and reported as a percentage of the control ± SEM.

### 2.7. RNA Extraction and cDNA Preparation

Hippocampal slices were lysed by adding 200 µL of TRI Reagent (Sigma-Aldrich, St. Louis, MO, USA) 24 h after LPS (1 μg/mL) treatment and stored at −20 °C until isolation. Total RNA was extracted using TRIzol^®^ Reagent according to the User Guide (Thermo Fisher Scientific, Waltham, MA, USA) based on the Chomczyński (1993) method [37]. After isolation, the RNA concentration was assessed using a NanoDrop spectrophotometer (ND/1000 UV/Vis, Thermo Fisher NanoDrop, Waltham, MA, USA) and the synthesis of cDNA was carried out via reverse transcription using an NG dART RT Kit (EURx, Gdansk, Poland) according to the manufacturer’s instructions.

### 2.8. Real-Time PCR

Real-time PCR was carried out using TaqMan probes and primers for: *Il-1β* (*Interleukin 1β*), *Tnf-a* (*Tumor necrosis factor α*), *Il-6* (*Interleukin 6*), *Cd40* (*Cluster of differentiation 40*), *CD68* (*Cluster of differentiation 68*), *Il-23a* (*Interleukin 23a*), *Igf-1* (*Insulin-like growth factor 1*), *Arg-1* (*Arginase 1*), *Il-4* (*Interleukin 4*), *Il-10* (*Interleukin 10), Il-13 (Interleukin 13), Cd74 (Cluster of differentiation 74), Ym1 (Chitinase 3-like-3 Chi3 l3/Ym1), Fizz (Found in inflammatory zone/resistin-like molecule*), *Il-27* (*Interleukin 27*), *Cx3cl1* (*Fractalkine*) and *Cx3cr1* (*Cx3c chemokine receptor 1*) (all obtained from Thermo Fisher Scientific, Waltham, MA, USA; Table 1) and the FastStart Universal Probe Master (Rox) kit (Roche, Basel, Switzerland) using the CFX96 Real-Time System (Bio-Rad, Hercules, CA, USA) as we described previously [36,38]. *Gapdh* (*glyceraldehyde 3-phosphate dehydrogenase*) (Thermo Fisher Scientific, Waltham, MA, USA) was used as an internal control for sample normalization.

### 2.9. Enzyme-Linked Immunosorbent Assays (ELISAs)

Twenty-four hours after LPS (1 μg/mL) treatment, OHC medium was collected to assess IL-1β, TNF-α, IL-6, IL-4, TGF-β and IL-10 levels. The protein levels of cytokines (ELISAs for interleukin 1β (IL-1β), interleukin 4 (IL-4), interleukin 6 (IL-6), interleukin 10 (IL-10), tumor necrosis factor α (TNF-α) and transforming growth factor β (TGF-β), all obtained from Cusabio, Houston, TX, USA) were measured in the culture medium using commercially available ELISA kits according to the manufacturers’ instructions.

Additionally, 24 h after treatment, the OHCs were lysed in accordance with the methodology described by us earlier [35,36]. Levels of the NLRP3 ((NACHT, LRR and PYD domains-containing protein 3 ELISA kit), NF-кB (nuclear factor-kappa B; both ELISA kits were from Cusabio, Houston, TX, USA), MyD88 (Myeloid differentiation primary response 88), CASP-1 (Caspase-1), PYCARD (Pyrin Domain-containing Protein 3, ELISA kit for PYD and CARD Domain-containing Protein), TRAF6 (TNF Receptor-associated Factor 6), GSDMD (Mouse Gasdermin D ELISA kit); all ELISA kits from ELK Biotechnology, Wuhan, China) and pNF-кB (NF-кB p65 (phospho) InstantOne ELISA kit, Thermo Fisher, Waltham, MA, USA) proteins in the OHCs lysates were measured using commercially available ELISA kits according to the manufacturers’ instructions. The detection limits were as follows: IL-1β < 7.8 pg/mL, IL-4 < 0.39 pg/mL, IL-6 < 0.39 pg/mL, IL-10 < 0.78 pg/mL, TNF-α < 3.9 pg/mL, TGF-β < 0.2 ng/mL, MyD88 < 0.061 ng/mL, CASP1 < 13.5 pg/mL, PYCARD < 3 pg/mL, TRAF6 < 0.53 ng/mL, NLRP3 < 1.56 pg/mL, NF-κB < 0.078 ng/mL, NF-κB p65 (phospho) not applicable and GSDMD < 0.063 ng/mL. The interassay precision of all ELISA kits was CV% < 10%. The intra-assay precision of all ELISA kits was CV% < 8%.

### 2.10. Statistical Analysis

Statistical analyses were performed using Statistica 10.0 software (Statsoft, Tulsa, OK, USA). All biochemical experiments were carried out under the same conditions for all samples, regardless of the type of treatment. The results presented in this study were derived from three independent KO FPR2−/− or WT OHCs, and the “*n*” for each culture was 2–5. All data were obtained in independent experiments and are presented as the means ± SEM (standard errors of the means). The results of the death processes (LDH) and NO release are presented as the mean percentages ± SEM of the control (vehicle-treated WT OHCs). The data obtained from the ELISA study are presented as the means ± SEM, and those for RT-PCR are presented as the average fold changes ± SEM. All groups were compared using a factorial analysis of variance (ANOVA) to determine the effects of the factors, followed, when appropriate, by Duncan’s post hoc test. In the case of the qRT-PCR analysis, factorial ANOVA was performed for those groups in which we obtained a value for measured factors. When appropriate, a two-way ANOVA was used. When the level of the tested factor was undetectable, a statistical analysis was not performed. A *p*-value < 0.05 was considered statistically significant. GraphPad Prism 5 was used for graphs preparation.

## 3. Results

### 3.1. Dynamics of OHCs Obtained from the Offspring of WT and KO Mice

**Figure 1:**
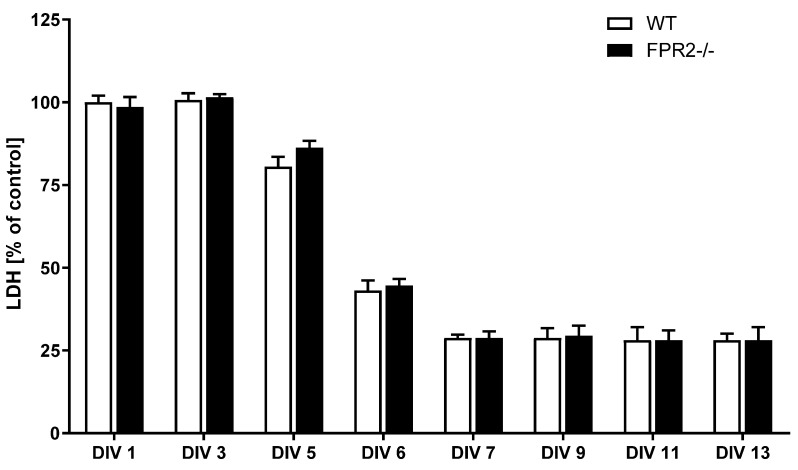
Dynamics of organotypic hippocampal cultures (OHCs) derived from 6–7-day-old offspring of wild-type (WT) and FPR2−/− (KO) mice. Dynamics of OHCs were assessed using the lactate dehydrogenase (LDH) assay. The data are presented as the mean percentages ± SEM of the control (vehicle-treated WT OHCs). LDH—lactate dehydrogenase; DIV—day in vitro.

In the first set of experiments, the dynamics of organotypic cultures were assessed based on the lactate dehydrogenase (LDH) release test. The LDH assay takes advantage of the release of the enzyme lactate dehydrogenase into the culture medium upon cell damage (disruption of the cell membrane). From the 7th day in vitro (DIV) of cultivation, the amount of LDH released into the medium remained at a low and constant level (until the 13th DIV) in both the WT and KO cultures (Figure 1). Based on this result, indicating a culture stabilization process, further experiments were carried out on the 7th day after culture stabilization.

### 3.2. The Effects of LPS and/or MR-39 on Cell Death and NO Release in OHCs Obtained from the Offspring of WT and KO Mice

**Figure 2:**
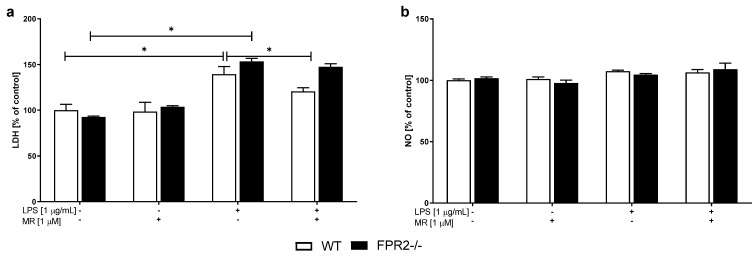
The effects of LPS and the compound MR-39 on cell death (**a**; LDH) and nitric oxide (**b**; NO) release in organotypic hippocampal cultures (OHCs) obtained from the offspring of wild-type (WT) and FPR2−/− (KO) mice. OHCs were pretreated with MR-39 and then stimulated with lipopolysaccharide (LPS; 1 μg/mL) for 24 h. Control cultures were treated with the appropriate vehicle. NO release was measured using the Griess reaction. The data are presented as the mean percentages ± SEM of the control (vehicle-treated WT OHCs) from three independent experiments. The results were statistically evaluated using a two-way analysis of variance (ANOVA) with the Duncan post hoc test to assess the differences between the treatment groups. Significant differences are indicated by * *p* < 0.05. LDH—lactate dehydrogenase; NO—nitric oxide. +—with LPS or MR treatment; -—without LPS or MR treatment.

First, we evaluated whether the new nonpeptidic agonist MR-39 exerted protective effects on LPS-evoked changes. We confirmed the harmful effect of LPS (1 μg/mL) on OHCs obtained from both WT (*p* = 0.014696) and KO (*p* = 0.001209) mice, as evidenced by an increased LDH level (Figure 2a). Interestingly, experiments performed on WT and KO cultures showed that pretreatment with MR-39 (1 μM) attenuated the LPS-induced increase in cell death processes, but only in WT (*p* = 0.013226) (Figure 2a) OHCs, which indicates the protective properties of this compound in the presence of FPR2.

We used an assay based on the Griess reaction to assess the effect of MR-39 on NO secretion from OHCs. OHCs were stimulated for 24 h, after which NO release was measured. As shown in Figure 2b, we did not observe an effect of either LPS (1 μg/mL) or MR-39 pretreatment on NO release in OHCs obtained from WT or KO mice.

### 3.3. The Effects of LPS and/or MR-39 on the mRNA Expression of Pro-Inflammatory and Anti-Inflammatory Factors in OHCs Obtained from the Offspring of WT and KO Mice

In the central nervous system, microglia are responsible for maintaining the physiological condition, while the shift from the M1- to M2-like profile is important in RoI. Generally, the M1-like phenotype is characterized by pro-inflammatory properties, while the M2-like phenotype is characterized by anti-inflammatory actions, which promotes tissue remodeling and repair. We decided to explore the effects of MR-39 on the pro- and anti-inflammatory phenotypes in OHCs from WT and KO mice under basal conditions and after LPS stimulation.

As shown in Table 2, the analyses of OHC samples revealed that LPS (1 μg/mL) significantly increased the expression of the *Il-1β* (*p* < 0.0001), *Tnf-α* (*p* = 0.020329), *Il-6* (*p* = 0.000542), *Cd40* (*p* = 0.028155) and *Il-23a* (*p* = 0.009358) mRNAs in the WT cultures. MR-39 treatment suppressed LPS-evoked increases in *Il-1β* (*p* < 0.0001) and *Il-6* (*p* = 0.001513) expression. In KO OHCs, a significant increase in the expression of the *Cd40* (*p* = 0.013854) mRNAs was observed. MR-39 treatment was not able to effectively modulate these changes.

Among the tested markers of the M2-like phenotype, the qRT-PCR analysis showed decreased expression of *Igf-1* (*p* = 0.043394), *Arg-1* (*p* = 0.039285), *Il-4* (*p* = 0.043573), *Fizz* (*p* = 0.038485) and *Cx3cr1* (*p* < 0.0001) after LPS (1 μg/mL) treatment in WT OHCs (Table 2). In contrast, LPS upregulated *Cd74* (*p* = 0.025336) expression. Interestingly, MR-39 reversed the changes in the expression of *Arg-1* (*p* = 0.002967), *Il-4* (*p* = 0.008535), *Cd74* (*p* = 0.048820), *Fizz* (*p* = 0.001408) and *Cx3cr1* (*p* = 0.03400) induced by LPS. On the other hand, in KO cultures, we did not observe any changes. The *Il-13*, *Ym1* and *Il-27* mRNAs were not detected in WT or KO OHCs (Table 2).

Additionally, the OHCs obtained from KO mice exhibited an altered microglial phenotype, since we noted abnormalities in the expression of genes related to both M1 and M2 phenotypes. The expression of the following factors was not detected: *Il-1β*, *Tnf-α*, *Il-6*, *Il-23a*, *Il-10*, *IL-13*, *Fizz* and *Il-27*. However, in the case of M1 phenotype-related factors, stimulation with LPS (1 μg/mL) increased the expression of *Cd40*.

### 3.4. The Effects of LPS and/or MR-39 on the Levels of Pro-Inflammatory and Anti-Inflammatory Factors in OHCs Obtained from the Offspring of WT and KO Mice

We assessed the anti-inflammatory and pro-resolving effects of the FPR2 agonist MR-39 on the production of the pro-inflammatory factors IL-1β, TNF-α and IL-6 and the anti-inflammatory factors IL-4, IL-10 and TGF-β in LPS-stimulated OHCs. Furthermore, to ensure that the observed effect was linked with interactions between ligand and FPR2, we pretreated cultures with the FPR2 antagonist WRW4.

**Figure 3:**
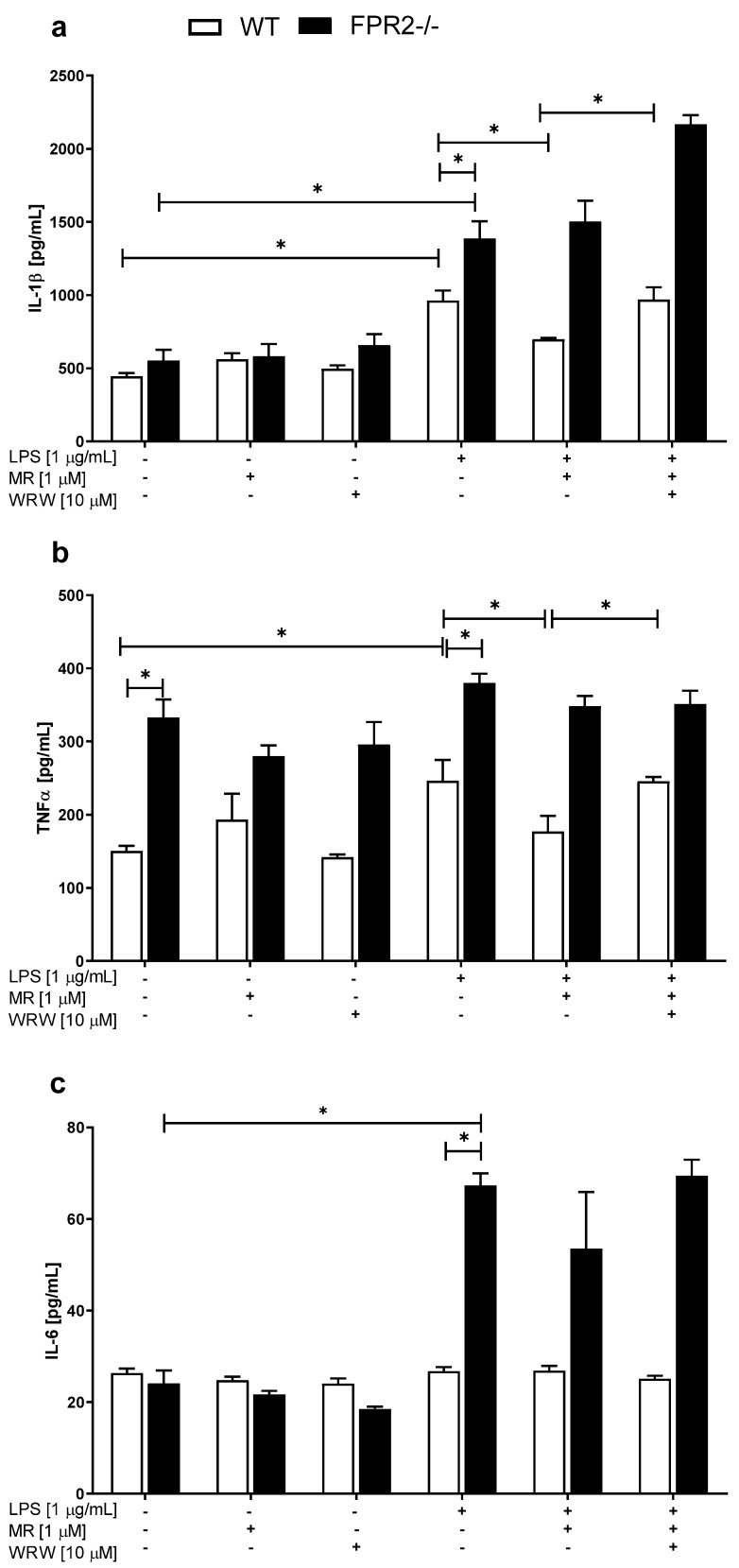
The effects of MR-39 and WRW4 on LPS-evoked pro-inflammatory cytokine release (**a**—IL-1β; **b**—TNF-α; **c**—IL-6) in organotypic hippocampal cultures (OHCs) derived from the offspring of wild-type (WT) and FPR2−/− (KO) mice. OHCs obtained from WT and KO mice were pretreated with the FPR2 antagonist WRW4 (10 µM) for 30 min. Afterward, MR-39 (1 µM) was added and incubated for 1 h, and then OHCs were stimulated with lipopolysaccharide (LPS; 1 μg/mL) for 24 h. Control cultures were treated with the appropriate vehicle. The results are presented as the means ± SEM. The data were obtained from three independent experiments. The results were statistically evaluated using a factorial analysis of variance (ANOVA) with Duncan’s post hoc test to assess the differences between the treatment groups. Significant differences are indicated by * *p* < 0.05. IL—interleukin; TNF-α—tumor necrosis factor α. +—with LPS, MR or WRW treatment; -—without LPS, MR or WRW treatment.

Stimulation with LPS (1 μg/mL) significantly increased IL-1β levels in the medium of the WT (*p* < 0.0001) and KO (*p* < 0.0001) cultures, as shown in Figure 3a. Experiments performed on WT and KO cultures showed that under basal conditions, neither MR-39 (1 μM) nor WRW4 (10 μM) altered IL-1β levels, as shown in Figure 3a. MR-39 (1 μM) treatment decreased (*p* = 0.028545) IL-1β levels only in WT hippocampal cultures stimulated with LPS (1 μg/mL), and pretreatment with the antagonist WRW4 was able to block the effect of MR-39 on IL-1β secretion (*p* = 0.030151). Interestingly, LPS (1 μg/mL) exerted a significantly greater on IL-1β release from the KO cultures than from the WT cultures (Figure 3a; *p* < 0.0001).

Moreover, in cultures obtained from KO mice, the level of TNF-α was higher than that in WT hippocampal cultures under basal conditions (Figure 3b; *p* < 0.0001). LPS (1 μg/mL) induced a significant upregulation of TNF-α production in the WT cultures (*p* < 0.02663) (Figure 3b). Importantly, a decrease in TNF-α secretion was observed after MR-39 (1 μM) treatment (*p* = 0.025743) only in WT hippocampal cultures, but was blocked by WRW4 (Figure 3b; *p* = 0.024152). These effects of MR-39 and WRW4 were not observed in the KO cultures.

We did not observe statistically significant changes in IL-6 levels between hippocampal cultures obtained from WT and KO mice (under basal conditions). LPS (1 μg/mL) induced a significant increase in the production of this cytokine only in the KO cultures (Figure 3c; *p* < 0.0001). Therefore, the LPS (1 μg/mL)-induced stimulation of IL-6 release was significantly higher in the KO cultures than in the WT cultures (Figure 3c; *p* < 0.0001).

**Figure 4:**
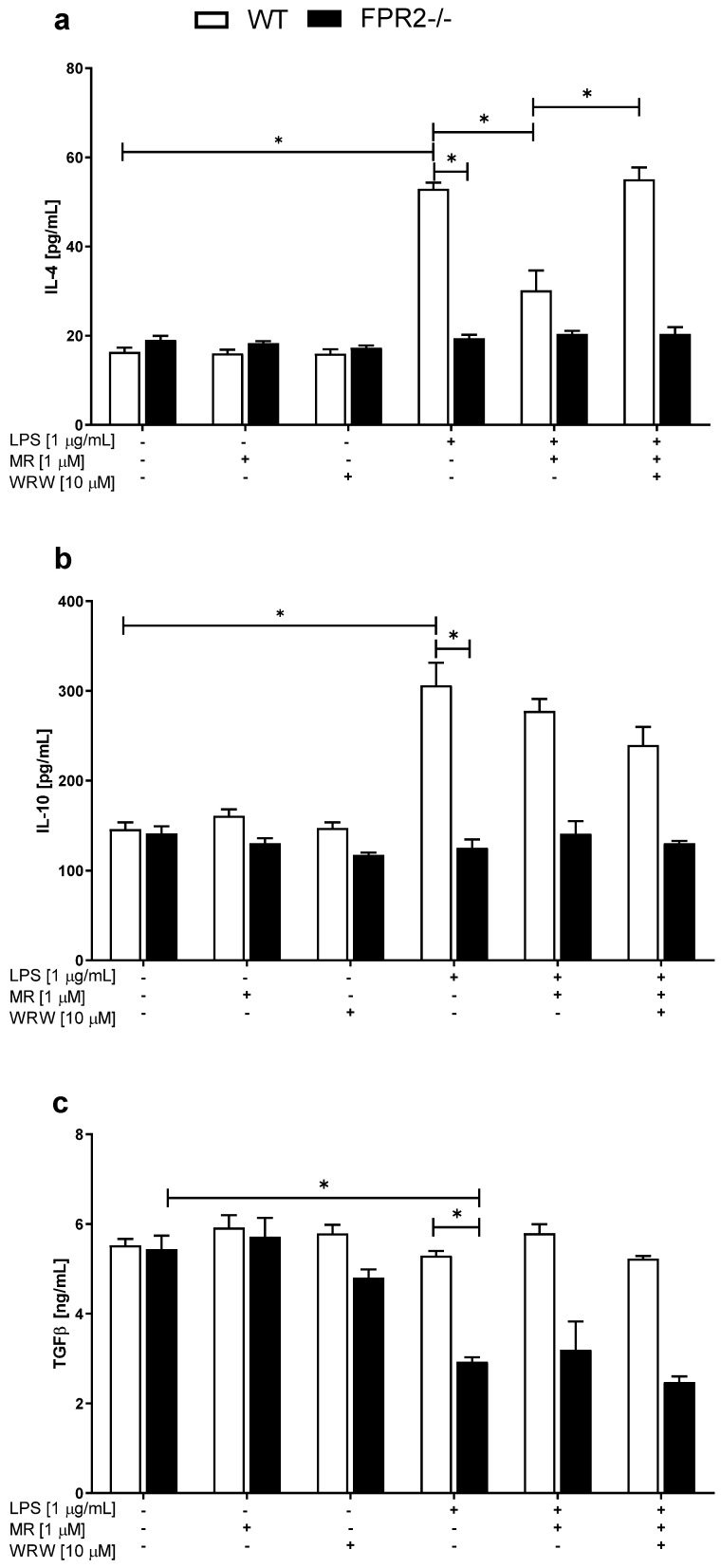
The effects of MR-39 and WRW4 on LPS-evoked anti-inflammatory cytokine (**a**—IL-4; **b**—IL-10; **c**—TGF-β) release in organotypic hippocampal cultures (OHCs) obtained from the offspring of wild-type (WT) and FPR2−/− (KO) mice. OHCs obtained from WT and KO mice were pretreated with the FPR2 antagonist WRW4 (10 µM) for 30 min. Afterward, MR-39 (1 µM) was added and incubated for 1 h, and then OHCs were stimulated with lipopolysaccharide (LPS; 1 μg/mL) for 24 h. Control cultures were treated with the appropriate vehicle. The results are presented as the means ± SEM. The data were derived from three independent experiments. The results were statistically evaluated using a factorial analysis of variance (ANOVA) with Duncan’s post hoc test to assess the differences between the treatment groups. Significant differences are indicated by * *p* < 0.05. IL—interleukin; TGF-β—transforming growth factor β. +—with LPS, MR or WRW treatment; -—without LPS, MR or WRW treatment.

Next, we measured the effects of LPS and/or MR-39 on anti-inflammatory cytokine levels. Experiments conducted on WT and KO OHCs demonstrated that under basal conditions, no changes were detected in the levels of IL-4 and IL-10 between the examined cultures (Figure 4a,b). LPS (1 μg/mL) treatment increased the levels of the IL-4 and IL-10 proteins only in WT cultures (*p* < 0.0001). In the case of IL-4, MR-39 suppressed the effect of LPS stimulation, while WRW inhibited MR-39 actions (Figure 4a,b; *p* < 0.0001; *p* < 0.0001). This effect was not observed on the KO cultures. Additionally, we did not observe effects of MR-39 on IL-10 levels. Meanwhile, the significant decrease in TGF-β release in KO hippocampal cultures evoked by LPS (1 μg/mL) (Figure 4c; *p* < 0.0001) was not modulated by the MR-39 treatment.

### 3.5. The Effects of LPS and/or MR-39 on TLR4-Related Pathways in OHCs Obtained from the Offspring of WT and KO Mice

One of the most important targets of LPS is TLR4, whose activation causes the upregulation of downstream proteins, such as the MyD88 adapter protein and transcription factors, including NF-κB, consequently leading to the synthesis of inflammatory genes. Additionally, TRAF6 is critical for both MyD88-dependent and MyD88-independent downstream signaling pathways mediated by NF-κB pro-inflammatory responses. Thus, we examined the effects of LPS and/or MR-39 on MyD88 and TRAF6 protein levels.

**Figure 5:**
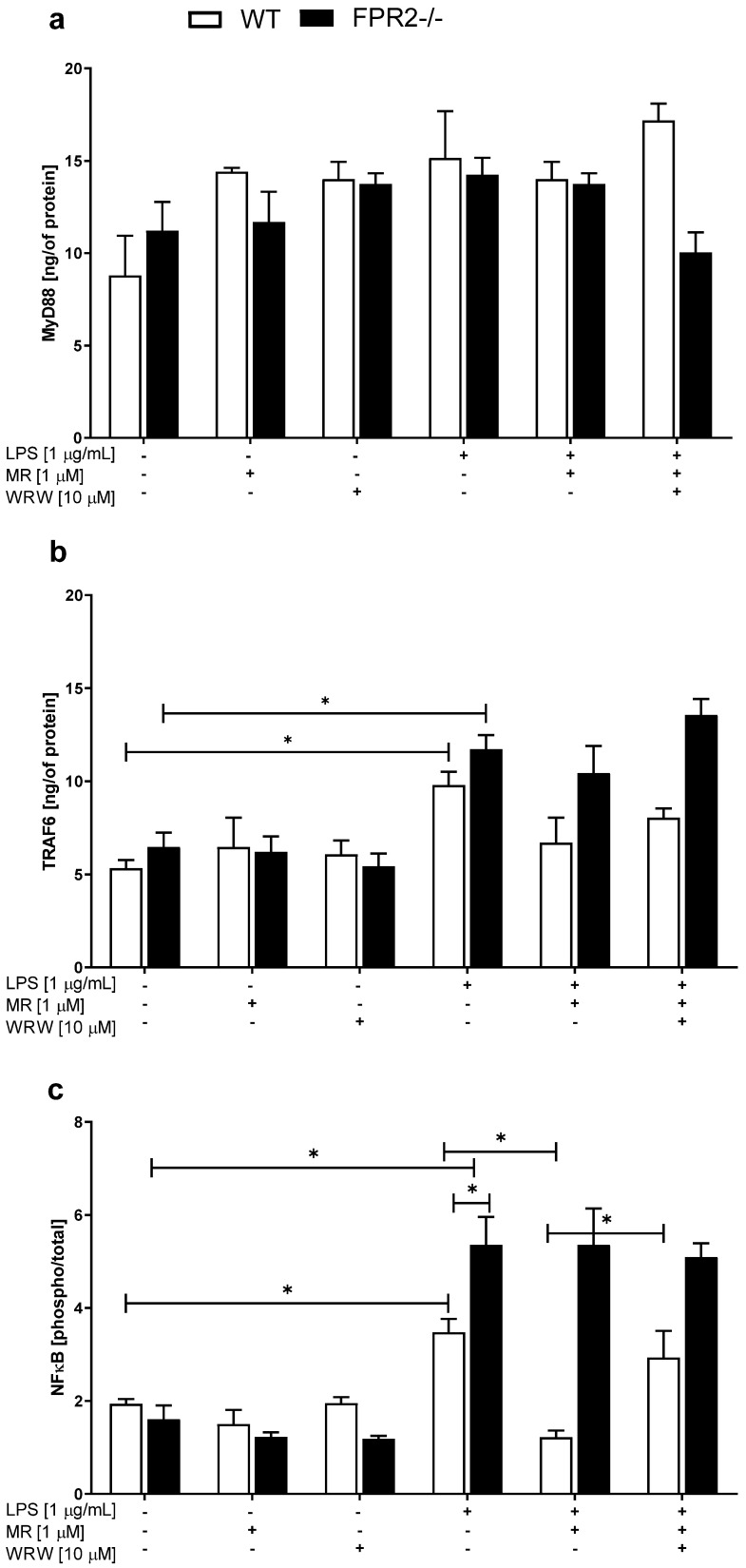
The effects of lipopolysaccharide and/or MR-39 and WRW4 on the (**a**) MyD88/(**b**) TRAF6/(**c**) NFκB-related pathways in organotypic hippocampal cultures (OHCs) derived from the offspring of wild-type (WT) and FPR2−/− (KO) mice. OHCs obtained from WT and KO mice were pretreated with the FPR2 antagonist WRW4 (10 µM) for 30 min. Afterward, MR-39 (1 µM) was added and incubated for 1 h, and then OHCs were stimulated with lipopolysaccharide (LPS; 1 μg/mL) for 24 h. Control cultures were treated with the appropriate vehicle. The results are presented as the means ± SEM. The data were obtained from three independent experiments. The results were statistically evaluated using a factorial analysis of variance (ANOVA) with Duncan’s post hoc test to assess the differences between the treatment groups. Significant differences are indicated by * *p* < 0.05. TRAF6—TNF receptor-associated factor 6; MyD88—Myeloid differentiation primary response 88; NFκB—Nuclear factor-κB. +—with LPS, MR or WRW treatment; -—without LPS, MR or WRW treatment.

None of the administered treatments affected the level of the MyD88 protein in the hippocampal cultures obtained from both WT and KO mice (Figure 5a). After LPS (1 μg/mL) stimulation, the level of TRAF6 was increased in cultures obtained from both WT (*p* = 0.018564) and KO (*p* = 0.04718) hippocampi (Figure 5b), but the MR-39 treatment was not able to effectively reverse these effects.

We did not observe any changes in the phospho-p65/total p65 ratio between WT and KO cultures under basal conditions. However, an increase in the phospho-p65/total p65 ratio was observed after LPS (1 μg/mL) stimulation in the WT (*p* = 0.015451) and KO cultures (Figure 5c; *p* < 0.0001). Moreover, the effect of LPS on the phospho-p65/total p65 ratio observed in the KO cultures was significantly stronger than that observed in the WT OHCs (*p* = 0.002216). Importantly, MR-39 only decreased the phospho-p65/total p65 ratio in the WT cultures (Figure 5c; *p* < 0.0001), while WRW4 blocked this effect (Figure 5c; *p* = 0.009803).

### 3.6. The Effects of LPS and/or MR-39 on the Levels of Proteins Involved in the NLRP3 Inflammasome Signaling Pathway in OHCs Obtained from the Offspring of WT and KO Mice

The NLRP3 inflammasome is a molecular platform within which inactive forms of the pro-inflammatory cytokines IL-1β and IL-18 are transformed into active forms. These cytokines are released from the cell to the extracellular space with the participation of NLRP3 to regulate the immune response in the central nervous system. Therefore, we estimated the effects of LPS and/or MR-39 on the protein levels of components of the NLRP3 inflammasome, such as NLRP3, caspase-1 and ASC.

**Figure 6:**
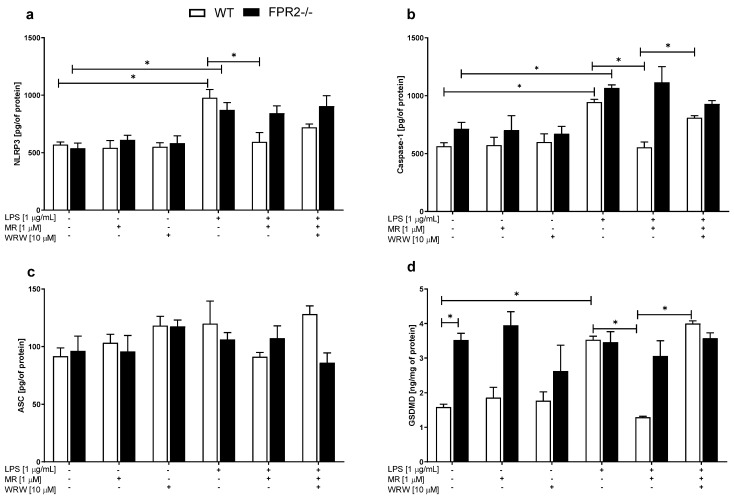
The effects of LPS and/or MR-39 and WRW4 on the levels of proteins involved in the NLRP3 inflammasome signaling pathway (**a**—NLRP3; **b**—Caspase-1; **c**—ASC, **d**—GSDMD) in organotypic hippocampal cultures (OHCs) derived from the offspring of wild-type (WT) and FPR2−/− (KO) mice. OHCs obtained from WT and KO mice were pretreated with the FPR2 antagonist WRW4 (10 µM) for 30 min. Afterward, MR-39 (1 µM) was added and incubated for 1 h, and then OHCs were stimulated with lipopolysaccharide (LPS; 1 μg/mL) for 24 h. Control cultures were treated with the appropriate vehicle. The results are presented as the means ± SEM. The data were obtained from three independent experiments. The results were statistically evaluated using a factorial analysis of variance (ANOVA) with Duncan’s post hoc test to assess the differences between the treatment groups. Significant differences are indicated by * *p* < 0.05. ASC—apoptosis-associated speck-like protein containing a caspase recruitment domain; NLRP3—Nod-like receptor pyrin-containing 3 subunit; GSDMD—gasdermin D. +—with LPS, MR or WRW treatment; -—without LPS, MR or WRW treatment.

No changes in the levels of examined proteins were observed under basal conditions in either of the examined groups (WT and KO). Stimulation with LPS (1 μg/mL) led to increased NLRP3 and Caspase-1 levels in both WT (*p* = 0.01030 and *p* = 0.002245, respectively) and KO (*p* = 0.04801 and *p* = 0.00353, respectively) hippocampal cultures (Figure 6a,b). Interestingly, MR-39 treatment effectively diminished the levels of the NLRP3 (*p* = 0.01402) and caspase-1 (*p* = 0.001881) proteins but only in WT hippocampal cultures (Figure 6a–c). Pretreatment with the antagonist WRW4 suppressed this effect on Caspase-1 (Figure 6b; *p* = 0.003693). On the other hand, none of the treatments affected the level of the ASC protein in the hippocampal cultures from the examined groups (Figure 6c).

Caspase-1 can also cleaves gasdermin D (GSDMD), which forms pores in the host cell membrane through which pro-inflammatory cytokines (IL-1β and IL-18) are released. On the other hand, GSDMD may induces the activation of the NLRP3 inflammasome and the associated proteolytic cleavage of pro-IL-1β and pro-IL-18. Thus, we also examined the effects of LPS and/or MR-39 on the GSDMD protein level.

In cultures obtained from KO mice, the level of GSDMD was higher than that in WT hippocampal cultures under basal conditions (Figure 6d; *p* = 0.046578). LPS (1 μg/mL) induced a significant upregulation of GSDMD production only in the WT (*p* = 0.048635) cultures. Importantly, after MR-39 (1 μM) pretreatment, the GSDMD level was decreased (*p* = 0.024790) in WT cultures, while this effect of MR-39 was blocked by WRW4 (Figure 6d; *p* < 0.0001). These effects were not observed on the KO cultures.

## 4. Discussion

The present study provided several lines of evidence that the new FPR2 agonist MR-39 exerts anti-inflammatory and cell-protective effects on an ex vivo model of neuroinflammation based on hippocampal organotypic cultures exposed to bacterial endotoxin.

Regarding the effects of the new FPR2 agonist MR-39, it inhibited LDH release and attenuated the OHC inflammatory status by affecting pro- and anti-inflammatory gene expression, as well as IL-1β, TNF-α and IL-4 release. Moreover, our biochemical data indicated that inhibition of the NLRP3 inflammasome pathways appears to play a key role in the anti-inflammatory mechanism of MR-39 action. The FPR2 specificity of MR-39 was supported by the ability of the antagonist, WRW4, to abrogate its actions, as well as by the absence of favorable MR-39 activity in hippocampal cultures derived from knockout mice.

In the present study, we employed organotypic hippocampal cultures (OHCs) derived from 6- to 7-day-old offspring of both WT and FPR2−/− KO mice. OHCs are innovative and reliable ex vivo models because they retain the neuronal-glia architecture and connectivity and permit studies of the effects of tested compounds on various cell types [39]. Furthermore, these ex vivo models enable pharmacological manipulations to investigate the mechanisms of inflammatory processes in the brain. FPR2 is expressed on microglia [40], astrocytes and hippocampal neurons [41]. OHC represents a particularly useful model for analyzing the role of new FPR2 ligands in the inflammatory response evoked by bacterial endotoxin.

In our research, we did not observe any changes in the dynamics/stabilization of OHCs obtained from WT and KO FPR2 mice or in lactate dehydrogenase (LDH) release evoked by LPS stimulation. As expected, MR-39 attenuated the LPS-induced increase of LDH levels in WT but not KO cultures. LDH is a stable cytoplasmic enzyme that is suddenly released into the cell culture medium because of cell membrane damages, and thus, it may be considered a key feature of cells undergoing apoptosis, necrosis and other forms of cell death [42]. Therefore, the inhibitory effect of MR-39 on LPS-induced LDH release in WT OHCs can be regarded as neuroprotective.

The endotoxin of gram-negative bacteria (lipopolysaccharide, LPS) is one of the most potent bacterial inducers of cytokine release, including the pro-inflammatory cytokines TNF-α, IL-1β and IL-6 [43], and gene expression of various other pro-inflammatory markers and factors. Consistent with these data, LPS increased the expression of *Cd40* in both WT and KO cultures. Moreover, as expected, in WT cultures, we observed upregulated expression of the *TNF-α*, *IL-1β*, *IL-6* and *IL-23a* mRNAs. Concurrently, MR-39 abolished the stimulatory effect of LPS administration on *IL-1β*, and *IL-6* expression but only in WT cultures. Furthermore, by exploring the effect of MR-39 on cytokine levels under basal conditions, we showed the upregulation of TNF-α synthesis in OHCs from KO FPR2 mice compared with WT cultures. Simultaneously, LPS treatment induced a more potent increase in the secretion of TNF-α from KO FPR2 hippocampal cultures. Similarly, IL-1β and IL-6 release were increased in KO FPR2 cultures after LPS treatment, potentially suggesting that OHCs obtained from KO mouse offspring have an altered ability to respond to inflammatory stimuli due to the lack of FPR2, which balances the response towards the resolution of inflammation [44]. This hypothesis appears to confirm the lack of the upregulation of various anti-inflammatory mediators, including *Ym-1*, *Cx3cr1*, *Il-10* and *Il-13*, as well as the release of the anti-inflammatory cytokines IL-10 and TGF-β in response to endotoxin stimulation observed in FPR2 KO hippocampal cultures. IL-10 exerts anti-inflammatory effects at least in part by regulating IL-1β production. Moreover, LPS specifically activates IL-10, triggering the induction of IL-10 secretion, which efficiently prevented pro-IL-1β expression. Thus, the equipoise between IL-10 induction and the amount of pro-IL1β potentially determines the final level of IL-1β [45]. IL-10 may also exert an anti-inflammatory effect through the fractalkine receptor (CX3CR1), which is mainly expressed in microglial cells [46,47]. Consistent with the results reported by Cunha et al. [48], in the present study, we observed that LPS diminishes *Cx3cr1* expression, but only in WT cultures. Thus, the upregulation of *Cx3cr1* mRNA expression by MR-39 in the presence of FPR2 might reinforce the regulatory effect of IL-10 on IL-1β production. Therefore, our study suggests that the lack of FPR2 affects the balance between pro- and anti-inflammatory responses in hippocampal cultures, shifting the profile of expressed factors and cytokines towards pro-inflammatory mediators.

Intriguingly, MR-39 only inhibited the pro-inflammatory response induced by LPS in the WT hippocampal cultures, as evidenced by increased TNF-α and IL-1β production, while it was blocked by WRW4 pretreatment. This observation indicates that the favorable effect of MR-39 was mediated by its interaction with FPR2, probably predominantly on microglial cells, which are the main source of IL-1β and TNF-α. Accordingly, we did not detect an effect of MR-39 on the increased level of IL-6 in KO FPR2 cultures.

In addition to the favorable effect of MR-39 on the pro-inflammatory cytokine profile, the data from the present study showed that the absence of FPR2 limited the effect of MR-39 on IL-4 release. In the brain, IL-4 is mainly produced by astroglial cells and induces both pro- and anti-inflammatory responses, depending on the treatment and timing paradigm. In some studies, IL-4 treatment reduced NO production and iNOS protein synthesis, as well as the secretion of TNF-α upon LPS stimulation [49]; thus, IL-4 elicits neuroprotective phenotypes in astrocytes [50]. In the current study, the upregulation of *Il-4* mRNA expression evoked by MR-39 pretreatment correlated at least in part with the upregulation of *Arg-1* and *Fizz* expression because *Il-4*-dependent M2 polarization of microglia is widely postulated [51]. Moreover, neurons from IL-4−/− mice were less effective than WT neurons at attenuating an inflammatory response, indicating that the absence of IL-4 increases the vulnerability to neuroinflammation. On the other hand, an IL-1β pretreatment of primary mouse astrocytes increased IL-6 production when cells were subsequently treated with IL-4 [52]. Moreover, long-term exposure to IL-4 induces MHCII expression in microglial cells [53]. Hence, the potential biological functions of IL-4 are complex and depend on the environment through mechanisms that are probably mediated by different processes in different tissues or conditions. Thus, the effect of IL-4 on OHCs in which both neuronal and glial cells are present should be considered complex, while the observed effect of MR-39 is ambiguous and requires further research.

The primary downstream signaling pathways affected by LPS include the MyD66/TRAF6/NF-кB pathway, whose activation leads to the synthesis of TNF-α, IL-1β and other inflammation-related factors [54]. Accordingly, in the next part of our study, we assayed the levels of proteins involved in the MYD88/TRAF6/NF-кB pathway in OHCs from both WT and KO FPR2 mice to provide more insights into the potential mechanism underlying the anti-inflammatory effect of MR-39. MR-39 had no effect on the MyD88 level and only tended to diminish the LPS-evoked increase in TRAF6 levels in WT hippocampal cultures. Nevertheless, the LPS treatment exerted a more pronounced effect on increasing the phospho-p65/p65 subunit ratio in KO FPR2 cultures compared with that in WT OHCs. We also found that the MR-39 treatment diminished the stimulatory effect of LPS on the NF-кB level only in WT cultures, and the WRW4 pretreatment blocked this effect of MR-39. Hence, we postulated that the anti-inflammatory activity of MR-39 toward TNF-α and/or IL-1β release was at least in part linked to the inhibition of the TRAF6/NF-кB pathway, while the presence of FPR2 was pivotal for this effect.

Growing pieces of evidence demonstrate that IL-1β is biologically inactive and must be cleaved and transformed into the bioactive form through the enzymatic activity of caspase-1. The proteolytic cleavage of procaspase-1 into active caspase-1 and maturation of IL-1β from its precursor form are triggered by NLRP3 inflammasome activation [55,56,57]. Activation of the NLRP3 inflammasome is strictly regulated. Indeed, its stimulation involves priming induced by the Toll-like receptor (TLR) and nuclear factor (NF-κB) [58]. Interestingly, activation of NF-κB evoked by lipopolysaccharide leads to the regulation of NLRP3 transcription by binding to NF-κB binding sites in the NLRP3 promoter [59,60]. Consequently, once primed, the following activation of the NLRP3 inflammasome, indicated as ‘the second signal,’ leads to the oligomerization of NLRP3 and the later assembly of NLRP3, ASC and procaspase-1 into a platform [61,62].

In the present study, LPS increased the levels of NLRP3 and caspase-1. We showed for the first time that MR-39 suppressed the increase in caspase-1 levels and that this effect was abolished by the WRW4 pretreatment, although only in WT cultures. This result suggests a possible association between FPR2 and the potential inhibitory effects of agonists of this receptor on NLRP3 pathways. In fact, annexin 1 (AnxA1), an agonist of FPR2, is required for IL-1β release in response to NLRP3 activators and is involved in NLRP3 inflammasome priming and assembly. Moreover, the absence of AnxA1 reduces the production and release of IL-1β in response to NLRP3 stimulation [63,64]. On the other hand, further studies are needed to determine whether this AnxA1 action depends on FPR2.

Notably, within the canonical pathway, the NLRP3 inflammasome serves as a platform for activating the proteolytic enzyme caspase-1, but this enzyme also processes gasdermin D (GSDMD) into a 30 kD fragment capable of oligomerizing and inserting in the plasma membrane, thus forming pores [65,66,67,68]. More advanced stages of GSDMD-mediated loss of membrane integrity result in pyroptosis. Nonetheless, membrane lysis and complete loss of its integrity are not an obstacle to the release of both pro-inflammatory cytokines—IL1β and IL-18—because of the presence of GSDMS pores. [69,70]. On the other hand, GSDMD may induce the activation of the NLRP3 inflammasome and the associated proteolytic cleavage of pro-IL-1β and pro-IL-18; thus, the GSDMD–NLRP3 interaction is complex and reciprocal.

Here, we documented increased GSDMD levels under basal conditions in KO FPR2 hippocampal cultures, which were affected neither by additional LPS stimulation nor by MR-39 treatment. However, LPS stimulation of WT hippocampal cultures increased GSDMD levels, which were reduced by the administration of MR-39, while WRW4 blocked these effects. This observation is in line with recent data pointing that LPS may directly targeting GSDMD [71]. We are fully aware that our present data have some methodological limitations (including the lack of evaluation of the caspase-1 activity and/or GSDMD cleavage); nonetheless, they shed more light on the MR-39 potential in the modulation of the NLRP3-related pathways in the inflammatory response.

## 5. Conclusions

Taken together, the results of the present study showed that the absence of FPR2 in hippocampal cultures leads to an aberrant inflammatory response to LPS stimulation and that the new FPR2 agonist MR-39 is a powerful inhibitor of some neuroinflammatory events. Moreover, our findings provide more insights into pathways inhibiting the NLRP3 inflammasome as a new molecular platform engaged in the protective effects of MR-39 on restraining inflammation in the brain. In our opinion, FPR2 offers a broad perspective for the development of promising new pro-resolving mediators, which deserve to be studied in further preclinical studies as candidates for the treatment of some brain disorders.

## Figures and Tables

**Table 1:** A list of genes (with corresponding catalog numbers of TaqMan probes) examined in organotypic hippocampal cultures (OHCs) that were obtained from the offspring of wild type (WT) and FPR2−/− (KO) mice using qRT-PCR. *Gapdh* was used as the reference gene.

Gene	Catalog Number
*Il-1β*	Mm00434228_m1
*Tnf-α*	Mm00443258_m1
*Il-6*	Mm00446190_m1
*Cd40*	Mm00441891_m1
*Il-23a*	Mm00518984_m1
*Cd68*	Mm03047343_m1
*Igf-1*	Mm00439560_m1
*Arg1*	Mm00475988_m1
*Il-10*	Mm01288386_m1
*Il-4*	Mm00445259_m1
*Il-13*	Mm00434204_m1
*Cd74*	Mm00658576_m1
*Ym1*	Mm00657889_mH
*Fizz*	Mm00445109_m1
*Il-27*	Mm04461162_m1
*Cx3cl1*	Mm00436454_m1
*Cx3cr1*	Mm00438354_m1
*Gapdh*	Mm99999915_g1

**Table 2:** The effects of LPS and/or MR-39, as well as WRW4, on mRNA expression in organotypic hippocampal cultures (OHCs) obtained from the offspring of wild-type (WT) and FPR2−/− (KO) mice. OHCs obtained from WT and KO mice were pretreated with MR-39 (1 µM) for 1 h and then stimulated with lipopolysaccharide (LPS; 1 μg/mL) for 24 h. Control cultures were treated with the appropriate vehicle. The results are presented as average fold changes ± SEM. The data were obtained from three independent experiments. The results were statistically evaluated using a factorial analysis of variance (ANOVA) with Duncan’s post hoc test to assess the differences between the treatment groups. * Indicates significant differences in mRNA expression compared to control (vehicle-treated) WT OHCs. ^#^ Compared to WT OHCs stimulated with LPS. ^ Compared to control (vehicle treated) KO OHCs. ND—Not detected.

Gene Expression								
**Factor**	**M1-Like Phenotype** **Wild-Type FPR2−/−**							
	**Control**	**LPS**	**MR**	**MR + LPS**	**Control**	**LPS**	**MR**	**MR + LPS**
*Il-1β*	0.85 ± 0.12	171.47 ± 15.98 *	3.26 ± 1.33	50.63 ± 16.41 ^#^	ND	0.80 ± 0.07	ND	0.80 ± 0.07
*Tnf-α*	1.06 ± 0.26	8.43 ± 0.50 *	1.73 ± 0.58	2.46 ± 0.58	ND	10.55 ± 0.6	ND	9.67 ± 3.95
*Il-6*	1.00 ± 0.01	1.49 ± 0.08 *	0.79 ± 0.05	1.07 ± 0.07 ^#^	ND	0.91 ± 0.14	ND	0.53 ± 0.03
*Cd40*	1.01 ± 0.09	1.98 ± 0.11 *	1.16 ± 0.11	1.26 ± 0.24	0.41 ± 0.10	1.53 ± 0.38 ^	0.80 ± 0.06	1.24 ± 0.13
*Il-23a*	1.00 ± 0.17	6.90 ± 3.23 *	0.63 ± 0.24	1.11 ± 0.03	ND	ND	ND	ND
*Cd68*	0.92 ± 0.01	0.81 ± 0.09	0.55 ± 0.09	0.58 ± 0.08	0.78 ± 0.29	0.39 ± 0.12	0.79 ± 0.15	0.33 ± 0.10
**Factor**	**M2-Like Phenotype** **Wild-Type FPR2−/−**							
	**Control**	**LPS**	**MR**	**MR + LPS**	**Control**	**LPS**	**MR**	**MR + LPS**
*Igf-1*	1.01 ± 0.14	0.51 ± 0.07 *	0.68 ± 0.17	0.44 ± 0.07	0.66 ± 0.21	0.34 ± 0.14	0.54 ± 0.15	0.24 ± 0.09
*Arg-1*	1.01 ± 0.10	0.46 ± 0.04 *	1.14 ± 0.21	1.35 ± 0.14 ^#^	0.88 ± 0.12	0.88 ± 0.10	0.71 ± 0.06	0.42 ± 0.10
*Il-10*	1.00 ± 0.07	0.31 ± 0.08	1.37 ± 0.44	3.32 ± 0.73	ND	ND	ND	ND
*Il-4*	1.02 ± 0.14	0.39 ± 0.08 *	1.37 ± 0.21	1.34 ± 0.15 ^#^	0.48 ± 0.02	ND	0.03 ± 0.10	ND
*Il-13*	ND	ND	ND	ND	ND	ND	ND	ND
*Cd74*	1.01 ± 0.11	4.44 ± 0.78 *	0.97 ± 0.13	0.85 ± 0.17 ^#^	0.38 ± 0.07	0.96 ± 0.11	0.87 ± 0.37	0.33 ± 0.07
*Ym1*	ND	ND	ND	ND	ND	ND	ND	ND
*Fizz*	1.03 ± 0.17	0.39 ± 0.07 *	1.18 ± 0.21	1.49 ± 0.29 ^#^	ND	ND	ND	ND
*Il-27*	ND	ND	ND	ND	ND	ND	ND	ND
*Cx3cl1*	1.02 ± 0.13	0.69 ± 0.06	1.44 ± 0.49	1.02 ± 0.07	0.45 ± 0.07	0.45 ± 0.07	0.49 ± 0.15	0.48 ± 0.09
*Cx3cr1*	1.03 ± 0.17	0.23 ± 0.02 *	0.72 ± 0.11	0.61 ± 0.11 ^#^	ND	0.30 ± 0.14	ND	0.15 ± 0.09

## Data Availability

All data supporting the conclusions of this manuscript are provided in the text, figures and tables.

## References

[1] DiSabato D.J., Quan N., Godbout J.P. (2016). Neuroinflammation: The devil is in the details. J. Neurochem..

[2] Guzman-Martinez L., Maccioni R.B., Andrade V., Navarrete L.P., Pastor M.G., Ramos-Escobar N. (2019). Neuroinflammation as a common feature of neurodegenerative disorders. Front. Pharmacol..

[3] Medzhitov R. (2010). Inflammation 2010: New Adventures of an Old Flame. Cell.

[4] Perretti M., Leroy X., Bland E.J., Montero-Melendez T. (2015). Resolution Pharmacology: Opportunities for Therapeutic Innovation in Inflammation. Trends Pharmacol. Sci..

[5] Corminboeuf O., Leroy X. (2015). FPR2/ALXR agonists and the resolution of inflammation. J. Med. Chem..

[6] Schwartz M., Baruch K. (2014). The resolution of neuroinflammation in neurodegeneration: Leukocyte recruitment via the choroid plexus. EMBO J..

[7] Fullerton J.N., Gilroy D.W. (2016). Resolution of inflammation: A new therapeutic frontier. Nat. Rev. Drug Discov..

[8] Basil M.C., Levy B.D. (2016). Specialized pro-resolving mediators: Endogenous regulators of infection and inflammation. Nat. Rev. Immunol..

[9] Regulska M., Szuster-Głuszczak M., Trojan E., Leśkiewicz M., Basta-Kaim A. (2020). The Emerging Role of the Double-Edged Impact of Arachidonic Acid- Derived Eicosanoids in the Neuroinflammatory Background of Depression. Curr. Neuropharmacol..

[10] Serhan C.N., Krishnamoorthy S., Recchiuti A., Chiang N. (2012). Novel Anti-Inflammatory-Pro-Resolving Mediators and Their Receptors. Curr. Top. Med. Chem..

[11] Martinez R.M., Fattori V., Saito P., Pinto I.C., Rodrigues C.C.A., Melo C.P.B., Bussmann A.J.C., Staurengo-Ferrari L., Bezerra J.R., Vignoli J.A. (2020). The Lipoxin Receptor/FPR2 Agonist BML-111 Protects Mouse Skin Against Ultraviolet B Radiation. Molecules.

[12] Yu Y., Ye R.D. (2015). Microglial Aβ Receptors in Alzheimer’s Disease. Cell. Mol. Neurobiol..

[13] Le Y., Oppenheim J.J., Wang J.M. (2001). Pleiotropic roles of formyl peptide receptors. Cytokine Growth Factor Rev..

[14] Migeotte I., Communi D., Parmentier M. (2006). Formyl peptide receptors: A promiscuous subfamily of G protein-coupled receptors controlling immune responses. Cytokine Growth Factor Rev..

[15] Bae Y.S., Song J.Y., Kim Y., He R., Ye R.D., Kwak J.Y., Suh P.G., Ryu S.H. (2003). Differential activation of formyl peptide receptor signaling by peptide ligands. Mol. Pharmacol..

[16] Horewicz V.V., Crestani S., De Sordi R., Rezende E., Assreuy J. (2015). FPR2/ALX activation reverses LPS-induced vascular hyporeactivity in aorta and increases survival in a pneumosepsis model. Eur. J. Pharmacol..

[17] Krishnamoorthy N., Abdulnour R.E.E., Walker K.H., Engstrom B.D., Levy B.D. (2018). Specialized proresolving mediators in innate and adaptive immune responses in airway diseases. Physiol. Rev..

[18] Sogawa Y., Ohyama T., Maeda H., Hirahara K. (2011). Formyl peptide receptor 1 and 2 dual agonist inhibits human neutrophil chemotaxis by the induction of chemoattractant receptor cross-desensitization. J. Pharmacol. Sci..

[19] Cooray S.N., Gobbetti T., Montero-Melendez T., McArthur S., Thompson D., Clark A.J.L., Flower R.J., Perretti M. (2013). Ligand-specific conformational change of the G-protein-coupled receptor ALX/FPR2 determines proresolving functional responses. Proc. Natl. Acad. Sci. USA.

[20] Ge Y., Zhang S., Wang J., Xia F., Wan J.B., Lu J., Ye R.D. (2020). Dual modulation of formyl peptide receptor 2 by aspirin-triggered lipoxin contributes to its anti-inflammatory activity. FASEB J..

[21] Chen T., Xiong M., Zong X., Ge Y., Zhang H., Wang M., Won Han G., Yi C., Ma L., Ye R.D. (2020). Structural basis of ligand binding modes at the human formyl peptide receptor 2. Nat. Commun..

[22] Chen K., Le Y., Liu Y., Gong W., Ying G., Huang J., Yoshimura T., Tessarollo L., Wang J.M. (2010). Cutting Edge: A Critical Role for the G Protein-Coupled Receptor mFPR2 in Airway Inflammation and Immune Responses. J. Immunol..

[23] Dufton N., Perretti M. (2010). Therapeutic anti-inflammatory potential of formyl-peptide receptor agonists. Pharmacol. Ther..

[24] Oldekamp S., Pscheidl S., Kress E., Soehnlein O., Jansen S., Pufe T., Wang J.M., Tauber S.C., Brandenburg L.O. (2014). Lack of formyl peptide receptor 1 and 2 leads to more severe inflammation and higher mortality in mice with of pneumococcal meningitis. Immunology.

[25] Maderna P., Cottell D.C., Toivonen T., Dufton N., Dalli J., Perretti M., Godson C. (2010). FPR2/ALX receptor expression and internalization are critical for lipoxin A 4 and annexin-derived peptide-stimulated phagocytosis. FASEB J..

[26] Schloer S., Hübel N., Masemann D., Pajonczyk D., Brunotte L., Ehrhardt C., Brandenburg L.O., Ludwig S., Gerke V., Rescher U. (2019). The annexin A1/FPR2 signaling axis expands alveolar macrophages, limits viral replication, and attenuates pathogenesis in the murine influenza A virus infection model. FASEB J..

[27] Serhan C.N., Savill J. (2005). Resolution of inflammation: The beginning programs the end. Nat. Immunol..

[28] Serhan C.N. (2007). Resolution phase of inflammation: Novel endogenous anti-inflammatory and proresolving lipid mediators and pathways. Annu. Rev. Immunol..

[29] Wang Y.P., Wu Y., Li L.Y., Zheng J., Liu R.G., Zhou J.P., Yuan S.Y., Shang Y., Yao S.L. (2011). Aspirin-triggered lipoxin A4attenuates LPS-induced pro-inflammatory responses by inhibiting activation of NF-κB and MAPKs in BV-2 microglial cells. J. Neuroinflamm..

[30] Yao C., Yang D., Wan Z., Wang Z., Liu R., Wu Y., Yao S., Yuan S., Shang Y. (2014). Aspirin-triggered lipoxin A4 attenuates lipopolysaccharide induced inflammatory response in primary astrocytes. Int. Immunopharmacol..

[31] Romano M. (2010). Lipoxin and aspirin-triggered lipoxins. Sci. World J..

[32] Stama M.L., Ślusarczyk J., Lacivita E., Kirpotina L.N., Schepetkin I.A., Chamera K., Riganti C., Perrone R., Quinn M.T., Basta-Kaim A. (2017). Novel ureidopropanamide based N-formyl peptide receptor 2 (FPR2) agonists with potential application for central nervous system disorders characterized by neuroinflammation. Eur. J. Med. Chem..

[33] Mastromarino M., Lacivita E., Colabufo N.A., Leopoldo M. (2021). G-Protein Coupled Receptors Involved in the Resolution of Inflammation: Ligands and Therapeutic Perspectives. Mini-Rev. Med. Chem..

[34] Stoppini L., Buchs P.A., Muller D. (1991). A simple method for organotypic cultures of nervous tissue. J. Neurosci. Methods.

[35] Basta-Kaim A., Ślusarczyk J., Szczepanowicz K., Warszyński P., Leśkiewicz M., Regulska M., Trojan E., Lasoń W. (2019). Protective effects of polydatin in free and nanocapsulated form on changes caused by lipopolysaccharide in hippocampal organotypic cultures. Pharmacol. Rep..

[36] Głombik K., Trojan E., Kurek A., Budziszewska B., Basta-Kaim A. (2019). Inflammatory Consequences of Maternal Diabetes on the Offspring Brain: A Hippocampal Organotypic Culture Study. Neurotox. Res..

[37] Chomczynski P. (1993). A reagent for the single-step simultaneous isolation of RNA, DNA and proteins from cell and tissue samples. Biotechniques.

[38] Chamera K., Kotarska K., Szuster-Głuszczak M., Trojan E., Skórkowska A., Pomierny B., Krzyżanowska W., Bryniarska N., Basta-Kaim A. (2020). The prenatal challenge with lipopolysaccharide and polyinosinic:polycytidylic acid disrupts CX3CL1-CX3CR1 and CD200-CD200R signalling in the brains of male rat offspring: A link to schizophrenia-like behaviours. J. Neuroinflamm..

[39] Sun X., Yao H., Douglas R.M., Gu X.Q., Wang J., Haddad G.G. (2010). InsulinPI3K signaling protects dentate neurons from oxygen-glucose deprivation in organotypic slice cultures. J. Neurochem..

[40] Cattaneo F., Guerra G., Ammendola R. (2010). Expression and Signaling of Formyl-Peptide Receptors in the Brain. Neurochem. Res..

[41] Ho C.F.Y., Ismail N.B., Koh J.K.Z., Gunaseelan S., Low Y.H., Ng Y.K., Chua J.J.E., Ong W.Y. (2018). Localisation of Formyl-Peptide Receptor 2 in the Rat Central Nervous System and Its Role in Axonal and Dendritic Outgrowth. Neurochem. Res..

[42] Kumar P., Nagarajan A., Uchil P.D. (2018). Analysis of cell viability by the lactate dehydrogenase assay. Cold Spring Harb. Protoc..

[43] Cavaillon J.M. (2018). Exotoxins and endotoxins: Inducers of inflammatory cytokines. Toxicon.

[44] Giebeler A., Streetz K.L., Soehnlein O., Neumann U., Wang J.M., Brandenburg L.O. (2014). Deficiency of formyl peptide receptor 1 and 2 is associated with increased inflammation and enhanced liver injury after LPS-stimulation. PLoS ONE.

[45] Sun Y., Ma J., Li D., Li P., Zhou X., Li Y., He Z., Qin L., Liang L., Luo X. (2019). Interleukin-10 inhibits interleukin-1β production and inflammasome activation of microglia in epileptic seizures. J. Neuroinflamm..

[46] Sheridan G.K., Murphy K.J. (2013). Neuron-glia crosstalk in health and disease: Fractalkine and CX3CR1 take centre stage. Open Biol..

[47] Chamera K., Szuster-Głuszczak M., Trojan E., Basta-Kaim A. (2020). Maternal Immune Activation Sensitizes Male Offspring Rats to Lipopolysaccharide-Induced Microglial Deficits Involving the Dysfunction of CD200-CD200R and CX3CL1-CX3CR1 Systems. Cells.

[48] Cunha C., Gomes C., Vaz A.R., Brites D. (2016). Exploring New Inflammatory Biomarkers and Pathways during LPS-Induced M1 Polarization. Mediat. Inflamm..

[49] Brodie C., Goldreich N., Haiman T., Kazimirsky G. (1998). Functional IL-4 receptors on mouse astrocytes: IL-4 inhibits astrocyte activation and induces NGF secretion. J. Neuroimmunol..

[50] Gadani S.P., Cronk J. (2013). Interleukin-4: A Cytokine to Remember. J. Immunol..

[51] Pepe G., Calderazzi G., De Maglie M., Villa A.M., Vegeto E. (2014). Heterogeneous induction of microglia M2a phenotype by central administration of interleukin-4. J. Neuroinflamm..

[52] Major J., Fletcher J.E., Hamilton T.A. (2002). IL-4 Pretreatment Selectively Enhances Cytokine and Chemokine Production in Lipopolysaccharide-Stimulated Mouse Peritoneal Macrophages. J. Immunol..

[53] Butovsky O., Talpalar A.E., Ben-Yaakov K., Schwartz M. (2005). Activation of microglia by aggregated β-amyloid or lipopolysaccharide impairs MHC-II expression and renders them cytotoxic whereas IFN-γ and IL-4 render them protective. Mol. Cell. Neurosci..

[54] El-Zayat S.R., Sibaii H., Mannaa F.A. (2019). Toll-like receptors activation, signaling, and targeting: An overview. Bull. Natl. Res. Cent..

[55] Mortellaro A., Diamond C., Khameneh H.J., Brough D. (2015). Novel perspectives on non-canonical inflammasome activation. Immuno Targets Ther..

[56] Kang H.J., Bae K.Y., Kim S.W., Kim J.T., Park M.S., Cho K.H., Kim J.M. (2016). Effects of interleukin-6, interleukin-18, and statin use, evaluated at acute stroke, on post-stroke depression during 1-year follow-up. Psychoneuroendocrinology.

[57] Swaroop S., Mahadevan A., Shankar S.K., Adlakha Y.K., Basu A. (2018). HSP60 critically regulates endogenous IL-1β production in activated microglia by stimulating NLRP3 inflammasome pathway. J. Neuroinflamm..

[58] Dowling J.K., O’Neill L.A.J. (2012). Biochemical regulation of the inflammasome. Crit. Rev. Biochem. Mol. Biol..

[59] Qiao Y., Wang P., Qi J., Zhang L., Gao C. (2012). TLR-induced NF-κB activation regulates NLRP3 expression in murine macrophages. FEBS Lett..

[60] Song N., Li T. (2018). Regulation of NLRP3 inflammasome by phosphorylation. Front. Immunol..

[61] Lamkanfi M., Kanneganti T.-D. (2010). Nlrp3: An immune sensor of cellular stress and infection. Int. J. Biochem. Cell Biol..

[62] Hanslik K.L., Ulland T.K. (2020). The Role of Microglia and the Nlrp3 Inflammasome in Alzheimer’s Disease. Front. Neurol..

[63] Galvão I., de Carvalho R.V.H., Vago J.P., Silva A.L.N., Carvalho T.G., Antunes M.M., Ribeiro F.M., Menezes G.B., Zamboni D.S., Sousa L.P. (2020). The role of annexin A1 in the modulation of the NLRP3 inflammasome. Immunology.

[64] Sanches J.M., Branco L.M., Duarte G.H.B., Oliani S.M., Bortoluci K.R., Moreira V., Gil C.D. (2020). Annexin A1 Regulates NLRP3 Inflammasome Activation and Modifies Lipid Release Profile in Isolated Peritoneal Macrophages. Cells.

[65] Aglietti R.A., Estevez A., Gupta A., Ramirez M.G., Liu P.S., Kayagaki N., Ciferri C., Dixit V.M., Dueber E.C. (2016). GsdmD p30 elicited by caspase-11 during pyroptosis forms pores in membranes. Proc. Natl. Acad. Sci. USA.

[66] Ding W., Guo H., Xu C., Wang B., Zhang M., Ding F. (2016). Mitochondrial reactive oxygen species-mediated NLRP3 inflammasome activation contributes to aldosterone-induced renal tubular cells injury. Oncotarget.

[67] Liu X., Zhang Z., Ruan J., Pan Y., Magupalli V.G., Wu H., Lieberman J. (2016). Inflammasome-activated gasdermin D causes pyroptosis by forming membrane pores. Nature.

[68] Sborgi L., Rühl S., Mulvihill E., Pipercevic J., Heilig R., Stahlberg H., Farady C.J., Müller D.J., Broz P., Hiller S. (2016). GSDMD membrane pore formation constitutes the mechanism of pyroptotic cell death. EMBO J..

[69] Evavold C.L., Ruan J., Tan Y., Xia S., Wu H., Kagan J.C. (2018). The Pore-Forming Protein Gasdermin D Regulates Interleukin-1 Secretion from Living Macrophages. Immunity.

[70] Heilig R., Dick M.S., Sborgi L., Meunier E., Hiller S., Broz P. (2018). The Gasdermin-D pore acts as a conduit for IL-1β secretion in mice. Eur. J. Immunol..

[71] Kayagaki N., Stowe I.B., Lee B.L., O’Rourke K., Anderson K., Warming S., Cuellar T., Haley B., Roose-Girma M., Phung Q.T. (2015). Caspase-11 cleaves gasdermin D for non-canonical inflammasome signalling. Nature.

